# Neutrophil gelatinase-associated lipocalin (NGAL) and tumor necrosis factor-α(TNF-α) levels in patients with schizophrenia

**DOI:** 10.1192/j.eurpsy.2023.582

**Published:** 2023-07-19

**Authors:** A. Gül Çakıl, H. Kaya, A. Sakallı Nural, I. B. Çakmak, I. T. Okay, E. Göka

**Affiliations:** ^1^Psychiatry, Bolu Mental Health Hospital, Bolu; ^2^Psychiatry, Ankara City Hospital, Ankara; ^3^Biochemistry, Hatay Training and Research Hospital, Hatay, Türkiye

## Abstract

**Introduction:**

Although the immune system is thought to contribute to the etiology of schizophrenia, the mechanism has not been clearly elucidated. Clarifying the relationship between them is important in terms of diagnosis, treatment, and prevention approaches.

**Objectives:**

In this study, it is aimed to determine whether there is any difference in serum levels of neutrophil gelatinase-associated lipocalin (NGAL) and tumor necrosis factor-alpha (TNF-α) in the group of patients with schizophrenia and healthy volunteers, whether these values are changed by medical treatment, whether there is any relation between these values and the severity of the symptoms of patients with schizophrenia, and whether NGAL can be used as a biomarker in the diagnosis and the follow-up of the schizophrenia.

**Methods:**

A total of 64 patients who were hospitalized in the Psychiatry Clinic of Xxxxxx and diagnosed with schizophrenia and 55 healthy volunteers were included in the study. A sociodemographic information form was given to all participants and TNF-α and NGAL values were measured. Positive and Negative Symptoms Rating Scale (PANSS) were applied to the schizophrenia group on admission and follow-up. TNF-α and NGAL levels were re-measured in the 4th week after the start of antipsychotic treatment.

**Results:**

As a result of the present study, it was found that NGAL levels decreased significantly after antipsychotic treatment of schizophrenia patients hospitalized with exacerbation (Figure 1). There was no significant correlation between NGAL and TNF-α levels among schizophrenia and the control group.

**Image:**

**Figure:**
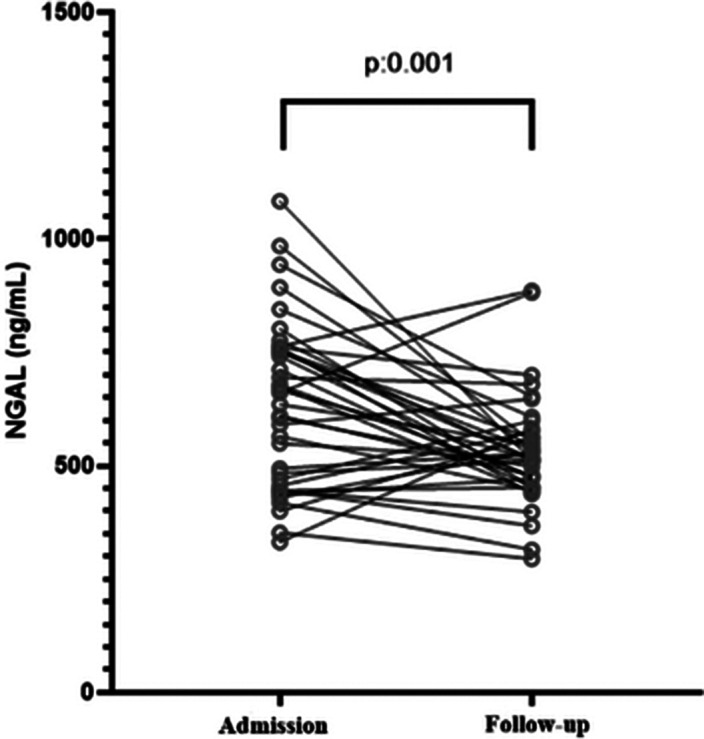

**Conclusions:**

In psychiatric diseases, especially schizophrenia, there may be differences in immune and inflammatory markers compared to the healthy population. After treatment, the NGAL levels of the patients at follow-up were reduced in comparison with the levels at their admission. It can be thought that NGAL may be related to psychopathology in schizophrenia and antipsychotic treatment. This is the first followup study for NGAL levels in schizophrenia.

**Disclosure of Interest:**

A. Gül Çakıl Grant / Research support from: This study funded by the Turkish Republic SBU Scientific Research Projects Coordinatorship. (Project no: 2020/013), H. Kaya: None Declared, A. Sakallı Nural: None Declared, I. Çakmak: None Declared, I. Okay: None Declared, E. Göka: None Declared

